# Effects of Organic Fertilizers on the Soil Microorganisms Responsible for N_2_O Emissions: A Review

**DOI:** 10.3390/microorganisms9050983

**Published:** 2021-05-01

**Authors:** Cristina Lazcano, Xia Zhu-Barker, Charlotte Decock

**Affiliations:** 1 Department of Land, Air and Water Resources, University of California Davis, Davis, CA 95616, USA; wyjzhu@ucdavis.edu; 2 Natural Resources Management and Environmental Sciences Department, California Polytechnic State University, San Luis Obispo, CA 93407, USA; cdecock@calpoly.edu

**Keywords:** N cycle, manure, compost, nitrification, denitrification, fertilizers

## Abstract

The use of organic fertilizers constitutes a sustainable strategy to recycle nutrients, increase soil carbon (C) stocks and mitigate climate change. Yet, this depends largely on balance between soil C sequestration and the emissions of the potent greenhouse gas nitrous oxide (N_2_O). Organic fertilizers strongly influence the microbial processes leading to the release of N_2_O. The magnitude and pattern of N_2_O emissions are different from the emissions observed from inorganic fertilizers and difficult to predict, which hinders developing best management practices specific to organic fertilizers. Currently, we lack a comprehensive evaluation of the effects of OFs on the function and structure of the N cycling microbial communities. Focusing on animal manures, here we provide an overview of the effects of these organic fertilizers on the community structure and function of nitrifying and denitrifying microorganisms in upland soils. Unprocessed manure with high moisture, high available nitrogen (N) and C content can shift the structure of the microbial community, increasing the abundance and activity of nitrifying and denitrifying microorganisms. Processed manure, such as digestate, compost, vermicompost and biochar, can also stimulate nitrifying and denitrifying microorganisms, although the effects on the soil microbial community structure are different, and N_2_O emissions are comparatively lower than raw manure. We propose a framework of best management practices to minimize the negative environmental impacts of organic fertilizers and maximize their benefits in improving soil health and sustaining food production systems. Long-term application of composted manure and the buildup of soil C stocks may contribute to N retention as microbial or stabilized organic N in the soil while increasing the abundance of denitrifying microorganisms and thus reduce the emissions of N_2_O by favoring the completion of denitrification to produce dinitrogen gas. Future research using multi-omics approaches can be used to establish key biochemical pathways and microbial taxa responsible for N_2_O production under organic fertilization.

## 1. Introduction

Organic fertilizers (OFs) are being strongly incentivized as a core management strategy to improve soil health and mitigate climate change by several regional and national initiatives. The use of OFs, such as compost and manure, constitutes a direct input of C to the soil, which can be stabilized through physical, chemical, and biochemical mechanisms [1] contributing to long-term storage of C in soils (i.e., C sequestration) [2,3,4]. A review of field studies showed that between 2–16% of the C applied through OFs was sequestered in soil over a long-term (i.e., more than 10 years) [5]. Organic fertilizers supply various C compounds with different chemical compositions, from labile to recalcitrant, that can be used by soil microorganisms during the process of mineralization to increase their growth rates and biomass. Thus, OFs have strong, short- and long-term effects on the soil microbiome and are fundamental to support soil health by increasing microbial activity, microbial interactions and nutrient cycling [6,7,8]. During the mineralization of C compounds in OFs, microorganisms also release plant-available nutrients. Therefore, OFs can indirectly increase soil C storage by increasing net primary productivity and root litter and exudation, a mechanism, which has been recently found to contribute to most of the sequestered or stable C in soils [9,10]. Additional benefits of OFs for climate change mitigation are related to the off-site reduction of greenhouse gas emissions associated with landfilling of organic wastes [11] and recycling of plant-available nutrients, including nitrogen (N), the most limiting nutrient for plant growth. Nonetheless, several fields and incubation studies showed that applying OFs resulted in large releases of greenhouse gases, most importantly nitrous oxide (N_2_O) [12,13]. The release of N_2_O can offset the climate change mitigation benefits of OFs, particularly when soil C sequestration potential has reached its plateau [14,15].

Nitrous oxide is a potent greenhouse gas with a global warming potential 298 times larger than carbon dioxide (CO_2_) and contributes to ozone depletion in the stratosphere [16]. The emission of N_2_O results from complex soil biotic and abiotic processes that produce and consume this greenhouse gas before its release into the atmosphere [17]. Nitrous oxide is produced in soils mainly as a byproduct or intermediate product of the microbial processes of nitrification and denitrification. These processes are strongly dependent on the availability of ammonium (NH_4_^+^), nitrate (NO_3_^−^) and labile C compounds and regulated by soil redox conditions, such as oxygen content and other soil physicochemical properties such as pH.

The use of fertilizers and manures in agricultural systems contributes to 23–31% of the global anthropogenic N_2_O emissions, most of which are released from soils after their application [18]. Organic fertilizers supply large amounts of inorganic N in both forms (i.e., NH_4_^+^ and NO_3_^−^) and, therefore, increase N_2_O production. Furthermore, organic fertilizers supply labile C compounds, which increase the activity of heterotrophic denitrifying microorganisms and trigger the emission of N_2_O [19,20,21,22]. The addition of OFs with high moisture content also promotes denitrification rates through changes in soil oxygen availability and redox conditions. Throughout the literature, it is observed that organic and inorganic fertilizers, when combined, resulted in higher N_2_O emissions per unit of N applied than if applied alone [12,23], showing that OFs have the potential to stimulate the release of N_2_O from previously applied inorganic N fertilizers. Nonetheless, several studies reported lower emissions from soil amended with organic than inorganic fertilizers or non-fertilized soils [24]. The production of N_2_O seems to differ depending on the type of OFs and their physicochemical properties, such as C: N ratio [25], with large differences, observed between plant-based vs. animal-based or composted vs. raw materials, as well as depending on soil conditions, such as soil texture, moisture, plant cover and quality [12,26]. Obviously, the effects of OFs on the microorganisms and processes of N_2_O emissions are far more complex than the effects produced by inorganic fertilizers.

Direct assessment of N_2_O emissions from soils is a challenge due to the extremely large spatial and temporal variability of the fluxes and the specialized analytical instrumentation required to detect and quantify this trace gas. Furthermore, accurately assessing N_2_O emissions requires high-frequency monitoring, which may become costly and time-intensive. Generally, rates of N_2_O production can reflect the changes in soil inorganic N pools. The relationship between N_2_O production, NH_4_^+^ and NO_3_^−^ has been established and used in the well-known hole-in-the-pipe model [27] to assess N_2_O emissions. In this model, the specific microbial process responsible for the production of N_2_O can be determined by assessing soil moisture levels, with denitrification being the predominant process under high soil moisture (>60% water-filled pore space). This model has been validated in several natural and managed ecosystems, including agricultural systems with inorganic fertilizers input [28]. In this model, the ratio of N_2_O produced to the inorganic N applied is used to calculate fertilizer emission factors. However, although N_2_O production is triggered by rapid changes in soil inorganic N pools and moisture, N_2_O production may ultimately depend on the capacity of the soil microbial community to adapt to these instant changes in environmental conditions and cycle N efficiently. Given that OFs impact soil microorganisms in many different ways, their effect on N_2_O production pathways, rates and dynamics are different from those of inorganic fertilizers and cannot be estimated solely through N inputs or changes in soil inorganic N pools [29]. For example, by providing C and N substrates to nitrifiers and denitrifiers, OFs may increase the size and activity of their communities, and therefore, increase N_2_O production rates; however, by increasing the diversity of these microorganisms, OFs may as well increase the consumption of N_2_O [30]. As a result, N_2_O emissions after using OFs may be better predicted by the size, diversity, structure, and/or functionality of the entire N-cycling microbial community, which would regulate the size of the “holes in the pipe”.

Currently, using isotopic tracers and molecular techniques allow for a finer resolution in the study of the microbial processes leading to the production of N_2_O. PCR-based analysis of ribosomal RNA marker genes allows for the taxonomic identification of N-cycling microorganisms in soils and quantifying the structure of the nitrifying and denitrifying microbial communities [30]. This same approach can be used to study the functionality of the microbial community by determining the abundance and expression of the main genes involved in the synthesis of enzymes that regulate the N cycle. Furthermore, high-throughput metagenomics and meta-transcriptomics allow for the parallel assessment of the presence and expression of different functional genes involved in the N cycle, providing a comprehensive understanding of the effects of different types of fertilization treatments on microbial N transformations [31]. However, at present, the abundance and function of N cycling microorganisms are seldom used to predict N_2_O production from soils. Thus, integrating microbial parameters into predictive models remains a knowledge gap.

A better knowledge of the microbial processes responsible for the production and consumption of N_2_O concerning the characteristics of OFs may help developing best management practices that optimize the benefits through improved soil health while reducing negative environmental impacts. If microbial data improves model performance, analysis of soil microbial community paired with the analysis of OF characteristics could be used to estimate N_2_O emissions in a more cost-effective way than the continuous monitoring approach. Furthermore, if there is a strong microbial fingerprint for N_2_O reduction associated with using OFs, a tool can be developed to verify greenhouse gas reductions in C markets. Currently, we lack a comprehensive evaluation of the effects of OFs on the function and structure of the N cycling microbial communities.

Here we provide an overview of the main microbial processes related to N_2_O production and summarize the current literature on assessing the effects of OFs on the abundance, diversity, community structure and function of nitrifying and denitrifying microorganisms in upland soils. We define organic fertilizers as naturally produced organic materials that are originated from different sources (e.g., animal and plant) and contain nutrients needed by plants. Organic fertilizers can be found in different degrees of decomposition or processing. Examples of unprocessed waste materials are raw manure, sewage sludge or industrial slurries of various types. Examples of processed materials are food waste hydrolysates, slaughter products (blood and feather meal), biochar, anaerobic digestates and composted materials. This review focuses on raw and processed animal manures with various physicochemical properties (i.e., plant-available N, C:N ratios). Based on the review, a framework of best management practices to minimize nutrient losses is proposed, and current challenges in research are discussed.

## 2. The N Cycle and Mechanisms Responsible for the Production and Consumption of N_2_O in Soils

**Figure 1:**
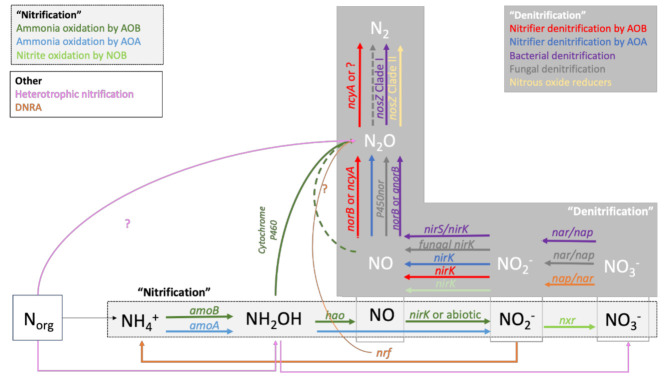
Processes and enzymes involved in N_2_O production. Figure adapted from [34,35]. For full enzyme names, see Table 1.

The hole-in-the-pipe model [32] was groundbreaking in conceptualizing how microbial processes drive N_2_O emissions and remain the backbone of major biogeochemical process models that predict N_2_O emissions from soil to date [33]. However, new developments in molecular techniques to study soil microorganisms have revealed a diversity of microorganisms involved in N_2_O emissions much more complex than previously thought (Figure 1).

Nitrification, referring to the oxidation of NH_4_^+^ to NO_3_^−^, was long assumed to be carried out by two distinct groups of autotrophic bacteria: ammonia-oxidizing bacteria (AOB) and nitrite-oxidizing bacteria (NOB). It also has been proved that ammonia-oxidizing archaea (AOA) can oxidize ammonia and that certain bacteria are capable of completing oxidation of NH_4_^+^ to NO_3_^−^, a process referred to as commammox [34]. A recently discovered cytochrome P460-mediated pathway explains the direct oxidation of NH_2_OH to N_2_O [35]. However, it is still unclear if this pathway only exists in AOB or if it also occurs in AOA. AOB is sensitive to low pH and favored in soils fertilized by single additions of high levels of inorganic NH_3_-based fertilizers, while AOA is dominant in acidic soils and grows preferentially when NH_4_^+^ is produced through mineralization of organic N [34].

Denitrification is traditionally known as the sequential reduction of NO_3_^−^ to N_2_ by heterotrophic denitrifying bacteria and fungi, where N_2_O is one of the intermediate products [36]. Denitrification is stimulated by high NO_3_^−^ concentrations, high availability of labile C, and low oxygen conditions. While stimulating denitrification and, therefore, N_2_O production, high labile C availability also stimulates the completion of denitrification and hence serves as a strategy to decrease N_2_O emissions through promoting N_2_O consumption [37]. The reduction of N_2_O to N_2_ is more sensitive to O_2_ exposure and low pH than the steps involved in reducing NO_3_^−^ to N_2_O. The enzyme *nosZ* responsible for this reaction is known to be inhibited at low soil pH [38,39]. Therefore, agricultural practices that create anaerobic microhabitats and increase pH can promote N_2_O consumption [40,41]. The denitrification process was traditionally thought to be completed within a single organism, namely heterotrophic facultative anaerobic bacteria [36]. Nevertheless, a recent study has shown that denitrification is widespread and found among diverse genera of bacteria, archaea, fungi and other eukaryotes [42]. Moreover, denitrification was shown to be a truncated process, with many organisms capable of carrying out only a few of the various steps involved in NO_3_^−^ reduction to N_2_ [42]. Denitrifying bacteria are found across several bacterial phyla, such as Proteobacteria, Nitrospira, Actinobacteria, Bacteroidetes, Firmicutes, Chloroflexi, Nitrospirae and Verrucomicrobia [42]. Archaeal denitrifiers belong to the phyla Euryarcheota, Thaumarcheota and Crenarcheota [42]. Denitrification is also a widespread trait in fungi, being present in several species within the order Hypocreales, Eurotiales, Sordariales, Chetosphaeriales, Mucorales, Pleosporales, Glomerellales and Ophiostomatales [43].

A relatively well-studied example of this widespread occurrence of denitrifying enzymes is the process referred to as nitrifier denitrification, defined as the reduction of NO_2_^−^ by ammonia oxidizers. This process may account for up to 100% of N_2_O emissions from NH_4_^+^ in soils and is more significant than classical denitrification under certain conditions [44]. Nitrifier identification is stimulated under NO_2_^−^ accumulation, while N_2_O production via hydroxylamine oxidation is likely triggered by high NH_3_ and low NO_2_^−^ in combination with high N oxidation rates [45]. Low O_2_ conditions favor nitrifier denitrification, but nitrifier denitrification may be more oxygen tolerant and less sensitive to pH change than classical denitrification [44]. The effect of C availability and source on nitrifier denitrification remains largely unknown [44]. An intriguing example of the truncated denitrification process is the recent discovery of a new clade of microorganisms that only possesses the denitrifying enzyme to reduce N_2_O to N_2_, raising the question of whether soil can be an effective sink of N_2_O rather than a source [46]. Factors that drive physiological and ecological responses of N_2_O reducing communities remain elusive. An improved understanding of niche differentiation between N_2_O producers and consumers could lead to innovative strategies to curb N_2_O emissions [42].

## 3. Methods to Study N_2_O Emissions and Underlying Processes in Soils

**Figure 2:**
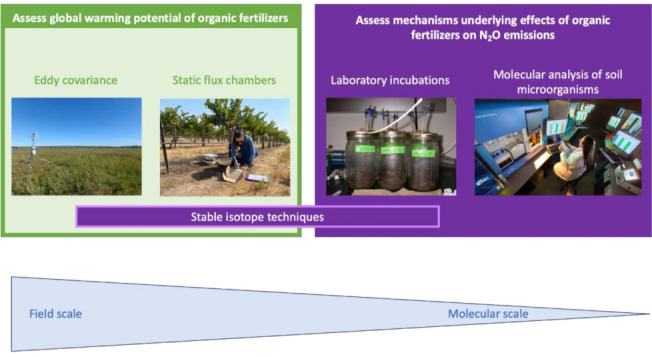
Methodologies used to study nitrous oxide emissions from soils across different scales.

N_2_O emissions from soil can be studied using several methods that operate at varying scales, depending on the research question to be addressed (Figure 2). When the objective is to assess the global warming potential of contrasting management practices, quantification of the annual N_2_O budget is essential. N_2_O fluxes from the soil are notoriously variable in space and time, characterized by short-lived emission pulses in several days following disturbances, such as precipitation, tillage or fertilization [47]. This necessitates data to be collected at high temporal and spatial resolution. Eddy covariance is a micrometeorological method that quantifies N_2_O fluxes semi-continuously over a footprint that can encompass a few hectares [48]. While this method overcomes the challenges associated with measuring the high temporal and spatial variable N_2_O fluxes, the methodology is impractical in replicated controlled experiments with relatively small plot size, which are most suitable for assessing the effect of different OFs under field conditions. In these scenarios, N_2_O emissions are most commonly assessed using static flux chambers. Static flux chambers, typically ranging in footprint from 0.03–1 m^2^, are installed in each treatment plot. Gas samples are collected manually or automatically during chamber closure, which typically lasts up to an hour. Nitrous oxide fluxes for each sampling date are calculated based on the increase in N_2_O concentrations over time during chamber closure [49]. Whether measurements are based on eddy-covariance or static flux chambers, quantifying N_2_O budget at a field scale is costly and time-consuming, limiting the number of treatment combinations that can be assessed. Given the large diversity of feedstocks and characteristics of OFs and their interaction with varying soil properties, this imposes important constraints on research aimed to assess the effect of OFs on N_2_O emissions.

Various soil incubation procedures have been used to study potential N_2_O emissions under laboratory conditions, including potential denitrification and denitrifier enzyme activity [50]. The procedures are rather poorly standardized across studies but do allow for comparisons within studies across a wider range of treatment combinations at a relatively low cost. Laboratory incubation studies are also commonly used to study mechanisms underlying N_2_O emissions. For example, soil can be incubated at varying moisture conditions to favor certain processes underlying N_2_O emissions, or selective inhibitors, such as acetylene or fungicides, can be used to block certain production or consumption pathways [51,52]. Mechanisms underlying N_2_O emissions can also be assessed using stable isotopes, either through using isotopically enriched tracer material or based on natural isotopic abundance, including the position of ^15^ N within the N_2_O molecule, referred to as site preference [53,54]. The enriched tracer method requires the addition of isotopically enriched substrate, which can be a challenge in the case of OFs. Isotopic natural abundance method can be employed at the laboratory or the field scale, but using the changes of isotopic signatures at the natural abundance level to distinct production pathways is a challenge as well due to the overlapping isotopic signatures among different N_2_O production and consumption processes.

The mechanistic understanding of N_2_O emissions following OF amendments can be enhanced by assessing the abundance of the microorganisms involved in N_2_O emissions. Over the last decade, studies have evaluated the effects of OFs on N cycling and N_2_O emissions by quantifying the abundance of the microorganisms involved in nitrification and denitrification via real-time quantitative PCR (qPCR) of marker genes or by measuring the abundances of the functional genes coding the enzymes involved in the production and consumption of N_2_O (Table 1) [55]. Similarly, the diversity of nitrifying or denitrifying microorganisms has been successfully studied using DGGE fingerprinting, high throughput sequencing of 16S rRNA or genes encoding N cycling enzymes [56].

Nitrifying soil organisms are typically studied based on RNA or DNA sequences of *amoA* genes encoding the enzyme ammonia monooxygenase [57,58]. Regarding denitrification, many enzymes are involved in the reduction of NO_3_^−^ to N_2_ (Figure 1). However, denitrifying microorganisms are most commonly studied based on the *nirK* and *nirS genes* encoding the nitrite reductases or *nosZ* genes encoding the N_2_O reductases. *NirK* is commonly observed in denitrifiers and nitrifiers, while *nirS* is thought to be exclusively found in denitrifiers [42]. It is important to note that bacterial, fungal and archaeal nitrifying and denitrifying enzymes require different primers to anneal properly with the target organisms’ DNA or RNA. Likewise, *nosZ* in the newly discovered clade of N_2_O reducers referred to as Clade II *nosZ* cannot be identified by primers designed for classical denitrifiers that possess Clade I *nosZ*. Studies that only include primers for Clade I *nosZ* or only assess bacterial DNA or RNA may gravely overlook the important changes in the structure, abundance and diversity of microorganisms that drive the response of N_2_O emissions to OFs.

## 4. Sources of Organic Fertilizers and N_2_O Emissions

Organic fertilizers are commonly used as nutrient sources in small-scale crop-livestock integrated systems. The use of OFs is an important practice to maintain or improve soil health and fertility in organic production systems where no synthetic nutrient sources are allowed [59]. Furthermore, in recent years, scientific evidence on the role of soil organic matter in soil health and climate change mitigation, together with an increasing emphasis on the circular economy, has reinvigorated the interest in using OFs in agriculture at large, including conventional and industrial large-scale crop productions. As a result, a small but steady increase in using organic waste-based fertilizers has been observed over the last two decades [60].

Confined animal feeding operations are the greatest sources of organic waste worldwide. Cattle manure is the animal waste produced in the largest amount in most countries, followed by poultry and pig manure [61]. Cattle manure production has increased steadily by 5%, as consumption of beef and milk increased globally. India is currently the largest producer of cattle manure, with 172 Tg y^−1^, followed by the USA (77 Tg y^−1^) and China (69 Tg y^−1^) [61]. Animal manures are sources of plant nutrients, such as organic and inorganic N. The total N excreted in animal manure globally ranges from 81.5 to 128.3 Tg y^−1^ [62]. Only in 2011, 120 Tg of manure N were excreted, an amount which was similar or larger than the total synthetic fertilizer N requirements worldwide [63]. Nevertheless, it should also be highlighted that the type and amount of N in animal manures vary largely. Manures are heterogeneous materials made of a mixture of animal feces and urine, which make up for different proportions of easily available N (urea, NH_4_^+^ and NO_3_^−^) and organic N (undigested protein, amino acids, urea and nucleic acids mostly). The actual proportion of inorganic and organic N depends on the animal species, livestock diet, N excretion rates, livestock bedding, and how the manure has been treated and processed [64,65]. For instance, the total N content in poultry manure is generally higher than in cattle (beef or dairy) or pig manure, while liquid manures have lower amounts of organic N and higher proportions of NH_4_^+^ when compared to solid manures [65]. For the same type of animal manure, bedding materials and manure processing and storage methods strongly influence the total amount and form of N, resulting in large differences between farm operations even in the same region [12]. For example, manure that has been processed aerobically through composting, vermicomposting or simply aging, contains relatively lower organic N and higher NO_3_^−^ than raw manure. On the other hand, manure that has been processed anaerobically, such as digester has relatively high organic N content with NH_4_^+^ dominates the inorganic N pool.

Despite the nutrient value of organic waste materials, it is well-known that mismanagement of OFs can lead to low N use efficiency and large losses to the environment, including the release of N_2_O after their application. A recent meta-analysis estimated that between 0.02 ± 0.13% and 1.21 ± 0.14% of the N in the applied OFs is emitted as N_2_O [29]. Depending on the usage and management of OFs, they can contribute to a significant portion of a region’s greenhouse gas budget. In India, it is estimated that 15,309 Gg CO_2_- C_eq_ per year are directly emitted as N_2_O from cattle manure, representing about 20% of the annual N_2_O emissions in the country. In the USA, cattle manure generates 6837 Gg CO_2_- C_eq_ as N_2_O per year, accounting for 2.2% of the total national emissions [61]. Nevertheless, throughout the literature, it is shown that emission factors can vary substantially depending on the physicochemical properties of the OF [29] and environmental conditions [66]. This suggests that a more detailed study on how OFs and their physicochemical properties affect soil microbial processes controlling N_2_O emissions is necessary.

## 5. Effects of Organic Fertilizers on Nitrifying and Denitrifying Soil Microorganisms

It is well known that the overall community structure of nitrifying and denitrifying microorganisms is strongly regulated by changes in substrate availability (NH_4_^+^, NO and NO_3_^−^), nutrient stoichiometry, labile C and soil physicochemical conditions, such as O_2_, pH or temperature [30,67]. By supplying inorganic N, C compounds and changing soil physicochemical properties, OFs can strongly shape the abundance and diversity of N cycling microorganisms in soils.

### 5.1. Effects of Raw Manure on the Structure and Activity of the Nitrifying and Denitrifying Community

Raw organic materials, such as animal manures, provide large inputs of NH_4_^+^ to a soil that directly impact the structure of the nitrifying community. NH_4_^+^ can also be released gradually through the mineralization of organic N in the manure after its application [31]. Ammonia oxidizers are particularly sensitive to the supply of NH_4_^+^ since they are lithoautotrophic microorganisms. Thus, animal manures typically increase the abundance and activity of ammonia oxidizers, leading to overall high nitrification rates [56,68]. While mineralization kinetics of organic N depends on environmental conditions and C:N ratios of the manure, nitrification of NH_4_^+^ proceeds relatively quickly, especially under high temperature and oxygen availability. As a result, all the easily available N can be nitrified within a few days to a few weeks, depending on soil texture and temperature, leading to subsequent peaks in N_2_O emissions [12,69].

The functional gene responsible for ammonia oxidation, the first step of nitrification, is *amoA*. The abundance of *amoA* gene copies is positively correlated with higher N_2_O emissions [70]. Higher nitrification rates lead to increased availability of NH_2_OH, NO and NO_3_^−^ (substrates for N_2_O production), as well as promote oxygen consumption, which can evoke denitrification and the subsequent release of N_2_O. In fact, several examples in the literature indicated increases in denitrification rates and abundance of the genes encoding for the denitrification enzymes after repeated application of raw manure (Table 2). Hallin et al. [71] reported that 50 years of cattle manure application increased the abundance of *narG* (encoding nitrate reductase), *nirK* and *nosZ* (encoding nitrite reductases) in Sweden. Similarly, in a laboratory incubation experiment, Cui et al. [72] observed that a soil that received pig manure for 30 years had a higher N_2_O emission baseline than an unfertilized control and an inorganically fertilized treatment; these higher emissions were positively correlated with the abundance of *nirK*, *nirS* and *nosZ*, the genes encoding nitrite and N_2_O reductases. Most likely, these high emissions were driven by the supply of organic C compounds that promoted microbial activity, consuming O_2_ and stimulating heterotrophic denitrification [73].

Raw animal feces often contain variable amounts of C, such as undigested cellulose, a relatively labile source of C [65]. Labile C generally constitutes up to 35% of the total C in manure [37]. Additionally, cattle and poultry are typically raised on concrete floors with bedding materials such as straw or woodchips. Therefore, manure is frequently mixed with variable amounts of bedding material which supplies additional C and contains compounds of higher recalcitrance like lignin. While highly recalcitrant C can reduce N_2_O emissions by promoting N immobilization, relatively labile sources of C such as cellulose stimulate the release of N_2_O by promoting denitrification [80]. Nevertheless, it has been observed that labile C in animal manure might promote the reduction of N_2_O to N_2_ by increasing the abundance of *nosZ* bearing bacteria, therefore, decreasing the overall emissions of this greenhouse gas [37]. The reduction of N_2_O to N_2_ is directly affected by soil pH since *nosZ* is inhibited by acidic soil conditions. Several studies have observed that the long-term application of inorganic fertilizers induces soil acidity, leading to increasing N_2_O emissions. Conversely, long-term application of organic fertilizers, such as manures, can reduce acidification and thus reduce N_2_O emissions [81].

Finally, another physicochemical parameter that drives N transformations in the soil is moisture. Liquid manures generally create anaerobic conditions rapidly and trigger N_2_O emissions [12]. In addition to the observed changes in microbial community function, animal manures change the structure and diversity of the nitrifying and denitrifying communities. Several studies showed that the dominance of nitrifying microorganisms switched towards ammonia-oxidizing archaea (AOA) after short- or long-term application of animal manures as it has been established that AOA is known to grow preferentially from mineralized NH_4_ [72,82,83]. However, this phenomenon seems to occur only in soils with low pH and substrate availability. In the soils with inorganic N additions or relatively high pH, AOB is still the main players [82,83], as reported by Lin et al. [68], who observed a shift from AOA to AOB accompanied by an increase in pH of an acidic soil after 44 years of pig manure and inorganic N additions. Microorganisms carrying *nirK* and *nirS* denitrifying genes are also sensitive to pH change and, as mentioned earlier, to changes in C availability [84]. Consequently, several studies showed that the structure of the denitrifying community is directly affected by manure inputs as compared to soils with no fertilizer inputs or with inorganic fertilizers [67,85]. Yin et al. [86] reported that 28 years of continuous application of pig manure significantly increased denitrification potential by increasing the abundance of *nirS* genes, then the same unfertilized soil, but not *nirK*. In a three-year field experiment, Huang et al. [67] observed that fertilization with cattle manure increased the abundance of *nirS*-harboring bacteria from the genera *Rubrivivax*, *Bradyrhizobium*, *Ideonella*, *Azoarcus* and *Polymophum,* resulting in significant changes in the overall structure of the soil-denitrifying community than the non-fertilized soil. These changes were significantly correlated to the C: N ratio of the manure, highlighting the importance of C in microbial denitrification.

### 5.2. Effects of Processed Manure on the Structure and Activity of the Nitrifying and Denitrifying Communities

Many approaches have been adopted to process organic waste materials, such as anaerobic digestion or pyrolysis, to obtain bioenergy, and composting or vermicomposting to produce biofertilizers. Often, waste materials are first processed through anaerobic digestion and followed up by composting, serving a dual purpose. These processes likely change the physicochemical properties of animal manures that have different impacts on soil microbiome and N_2_O emissions.

Anaerobic digestion is the degradation of organic substrates by a consortium of anaerobic microorganisms [87]. Both solid and liquid manure can be processed through anaerobic digestion to generate biogas (a mixture of CH_4_ and CO_2_) and digestate. Digestates are characterized by the lower amount of available C, higher relative amounts of recalcitrant compounds (such as lignin or non-hydrolyzable lipids), and higher NH_4_^+^: total N ratio as compared to the initial manure [88,89]. This high amount of NH_4_^+^, together with the high pH that is generally found in digestates, increases the risk of ammonia volatilization, a major concern for N losses and human health [89]. On the other hand, the literature shows contrasting data on the effects of anaerobic digestates on soil N_2_O emissions as compared to raw manure (Table 2). For example, in an incubation study, Cayuela et al. [90] observed that digestates produced from cattle or pig manure resulted in lower soil N mineralization rates and N_2_O emissions than the initial feedstocks. Similar results were observed by Grave et al. [91] in a field study comparing raw and anaerobically digested pig slurry, and the differences were attributed to the low content of volatile solids and labile C compounds in the digested slurry. In contrast, Saunders et al. [74] observed that in a laboratory incubation, cumulative soil N_2_O emissions were more than five times higher in the digestate than the raw cattle manure treatments. These contrasting results seem to be directly correlated to the physicochemical properties of the digestates, most importantly the contents of available C and NO_3_^−^. Saunders et al. [74] showed that in the soils amended with digestate, higher N_2_O emissions were generally correlated with higher abundances of *amoA*, *narG*, and *nosZ* and high N substrate availability in the digested manure, compared to raw manure.

Composting promotes the accelerated microbial decomposition of organic waste materials under aerobic conditions. Composting, which can take up to several months, is characterized by an initial 2-week thermophilic stage, where decomposition of labile compounds proceeds at very high rates [92]. During this phase, heat is generated, which allows for the sanitization of the waste material. Subsequently, the amount of labile compounds decreases and the microbial decomposition slows down, and the composting enters into the maturation phase [93]. As a result, compared with the initial feedstock, the final product of composting (i.e., compost) is more stable with a lower proportion of labile organic C and N compounds, higher NO_3_^−^/NH_4_^+^ ratio, and lower C:N ratio [93]. Accordingly, composted manure typically has different effects on the structure and function of the soil microbiome than raw manure [7,94]. Compost application to soils has been shown to induce short-term increases in the abundance of nitrifying and denitrifying microorganisms, resulting in N_2_O production [30]. Similarly, long-term application of compost increases the abundance of ammonia-oxidizing bacteria and denitrifying microorganisms (*nosZ*, *nirK*, *nirS*) than inorganic fertilizers (Table 2) [75]. Yet, N_2_O emissions from composts were comparatively lower than raw manures due to a lower input of easily available organic C and slower nitrification and denitrification rates associated with compost compared to raw manure [29,95]. Nevertheless, the addition of composted swine manure (rich in available organic N and recalcitrant C) to a clay loam inceptisol had similar cumulative N_2_O emissions as the untreated swine slurry. This shows that the differences in N_2_O emissions between raw and composted manure may be dependent on the type of manure, especially the availability of dissolved organic N and C in the manure [24].

Similar to compost, vermicompost is a stabilized organic fertilizer with a lower proportion of labile organic C and N compounds, higher NO_3_^−^/NH_4_^+^ ratio, and lower C:N ratio compared to raw manure [92]. During the vermicomposting process, earthworms graze on microorganisms, shaping the structure of the microbial community and increasing nitrification rates [96]. Typically, soils amended with vermicompost have a different microbial community structure than soils amended with raw or composted manures, including differences in AOB [6,97]. In a column incubation experiment, Liu et al. [78] observed that applying vermicompost amendment to a saline-alkaline soil increased nitrification rates by increasing archaeal and bacterial *amoA* gene copy numbers than a non-amended control. Nevertheless, nitrite-reducing bacteria were inhibited by the vermicompost, as shown by the decreased *nirS* and *nirK* gene copy numbers after its application. This, together with the increase in *nosZ* gene copies, suggests that using vermicompost may not lead to large N_2_O emissions, compared to raw manure or other organic amendments. Rodriguez et al. [21] reported that soil amended with vermicomposted biosolids produced less N_2_O emissions than soil with raw biosolids, although this depended on the moisture: at high soil moisture, N_2_O emissions were high regardless of the type of soil amendments.

Another alternative for the valorization of animal manure is combustion through pyrolysis to produce energy and biochar. Biochar produced from manure is a source of stable C that contributes to long-term C sequestration and improvements in soil fertility due to its nutrient content, high surface area, porosity and high pH, although production temperature can cause substantial variability in these physicochemical properties [98]. Due to its chemical stability, manure biochar typically results in lower N_2_O emissions than raw or composted manure [99]. Biochar also influences N_2_O emissions indirectly by changing the amount of inorganic N available in the soil. Typically, the high adsorption capacity of biochar reduces inorganic N availability and N_2_O production. Nevertheless, between 1 and 20% of the organic N in manure biochar can be mineralized in the short term and subsequently increase the nitrification rate. Biochars with lower aromaticity also have higher amounts of available C that can increase denitrification rates (*nirK*, *nirS*, and *nosZ*) and N_2_O emissions [79] (Table 2). Additionally, the observed effects are dependent on biochar interactions with soil texture, pH, moisture and soil N [100,101]. N_2_O emissions tend to be higher in acidic, coarse-textured soils at high soil moisture or in fine-textured soils at low moisture conditions [100]. Finally, increases in soil pH induced by the typically alkaline biochars can induce changes in the dominance and diversity of nitrifying and denitrifying microorganisms [98].

### 5.3. Management Strategies to Reduce N_2_O Emissions from Soils Amended with Manure-Based Organic Fertilizers

As mentioned earlier, the microbial processes that lead to the production of N_2_O in soils are largely regulated by substrate availability. Thus, regulating N_2_O emissions requires tight control of inorganic N in soils. The 4R of fertilizer management is a decision-making tool that helps growers to reduce nutrient (mainly N) losses. The 4R strategy uses information on crop N requirements, fertilizer and soil N content to establish the right N source, the right application rate, right timing and placement. Following the 4R principles, a careful evaluation of the amount of inorganic N provided by a specific OF is necessary to reduce N_2_O emissions. Furthermore, the amount of labile C also needs to be taken into account due to its role in stimulating denitrification. The availability of N and C depends on the type of animal manure but also on the processing approach used. Generally, raw manures contain high amounts of available C and N than processed manures (compost, vermicompost and digestates) and should be managed carefully to avoid evoking emissions [13]. Calculating the right rate of fertilizer requires adjusting inputs to satisfy plant needs taking into account the nutrients already available in the soil. For raw manures, the right application rate is sometimes calculated based on P and K rather than N due to the larger amount of these two plant macronutrients in the manure relative to the plant’s needs and the risk of overapplication. Yet, care should be taken to match the addition of large amounts of immediately available N with plant needs. It is expected that, in addition to the immediately available inorganic N, a substantial amount of N in OFs will become available to the crop through the mineralization of organic compounds. Thus, to calculate the right rate of OFs, immediately and potentially available N (PAN) need to be both taken into consideration [102].

Biochar, with its high surface area and adsorptive capacity, has the potential to reduce the amount of available N in manure-based OFs and subsequently lower N_2_O emissions. Yuan et al. [103] observed that mixing composted manure with biochar reduced denitrifier abundance, as shown by lower *nirK* abundances and resulted in lower N_2_O emissions compared with the composted manure alone. Several recent studies showed that inorganic N availability and N_2_O emissions from OFs could also be effectively reduced through the use of nitrification inhibitors. Nguyen et al. [104] showed that 3,4-dimethyl pyrazole phosphate reduced the N_2_O emissions from the soil after the application of cattle manure by 60%. Vallejo et al. [24] reported that the nitrification inhibitor dicyandiamide reduced N_2_O emissions from soil amended with pig manure and composted pig manure by 83% and 77%, respectively. Nevertheless, Duan et al. [105] suggested that nitrification inhibitors are not as efficient in reducing N_2_O emissions as biochar since they may stimulate denitrification.

A good understanding of the fertilizer mineralization temporal dynamics is also required to match nutrient release with crop needs and optimize nutrient use efficiency. Processed manures, such as compost, have a higher proportion of stable organic compounds that provide a gradual and steady release of N. Thus, a single application at pre-plant is recommended to allow enough time for these OFs to release mineral N [106]. On the other hand, to avoid large emissions of N_2_O, split applications are recommended for certain unprocessed manure-based OFs with high concentrations of inorganic N, such as liquid manure [12]. Lastly, research showed that N_2_O emissions are also affected by the manure placement method. Depending on the cropping system, manure can be applied to the soil surface, incorporated or injected into the sub-surface. N_2_O emissions are typically higher when manure is injected into soils than surface application, due to higher chances of contact between manure and soil microorganisms that produce N_2_O, as well as creating anaerobic conditions at the injection sites, which are further enhanced by high soil moisture [107]. Nevertheless, Velthof et al. [13] observed that manure N_2_O emissions decreased with injection depth, which was attributed to the larger diffusion path and the larger chance for the produced N_2_O to be reduced to N_2_ while transported to the soil surface.

In addition to controlling N inputs, N availability can be managed through practices that promote microbial N immobilization and tighten the N cycle. Agroecological management and the overall improvement of soil health through long-term compost application can reduce soil N losses such as N_2_O emissions [108,109]. Several studies showed that the repeated application of composted manure induced changes in soil physicochemical properties and microbial communities, which may help reduce emission peaks in response to the pulses of available N and C associated with a single fertilizer application. Long-term repeated compost additions increase the diversity, activity and abundance of denitrifiers (as shown by higher *nirS*, *nirK*, and *nosZ* gene abundance), allowing for completed denitrification to proceed and resulting in comparatively lower N_2_O emissions than the soils without any compost inputs [70,76,77]. It has been suggested that C accumulation in soils due to compost inputs allows for denitrification to be completed, and promotes the immobilization of surplus N, therefore, reducing N_2_O emissions [110]. Identification of the microbial species responsible for N_2_O reduction under long-term compost additions would open the door to microbiome engineering. The abundance and/or presence of these species could be used to improve predictions of N_2_O emissions under organic fertilization and verify greenhouse gas reductions in C markets.

The promotion of beneficial plant-microbe interactions is also a fundamental component of agroecological management that contributes to improving plant nutrient use efficiency and decreasing N losses. For instance, symbiosis with arbuscular mycorrhizal fungi (AMF), a widespread fungal symbiont capable of colonizing most terrestrial plants, increases plant N and water uptake, therefore, indirectly driving soil N and moisture changes. As a consequence, AMF colonization typically reduces N_2_O emissions [111] and has a strong impact on N_2_O^−^ related microorganisms, such as reducing *nirK* and increasing *nosZ* gene abundances [112]. No-till or reduced-till promotes C sequestration and the AMF colonization [113], thus potentially promoting various pathways to mitigate N_2_O emissions. Nevertheless, several studies showed higher nitrification and denitrification rates and N_2_O emissions in no to than tilled soils in the first 10 years following practice conversion [91,114], which were attributed to the increased water-filled pore space and N availability than tilled soils [91,115].

## 6. Conclusions, Future Directions and Research Needs

Organic fertilizers have strong impacts on the structure and function of nitrifying and denitrifying microorganisms. Several fields and incubation studies show increases in the abundance of genes responsible for nitrification (*amo*) and denitrification (*narG, nirS, nirK, nosZ*) after applying organic fertilizers, which result in N_2_O emissions. These changes in soil microorganisms are related to the supply of N substrates for nitrification and denitrification, the supply of labile C that stimulates heterotrophic denitrification, and the modification of soil conditions (e.g., change pH and oxygen availability).

Nevertheless, the large heterogeneity in the physicochemical properties of OFs due to the different feedstocks and processing methods results in extremely variable effects on soil microorganisms and N_2_O emissions. Typically, unprocessed manure with high moisture content can produce a short-term increase in N_2_O emissions, especially when plant N demand is low. Nonetheless, processed and highly stable OFs, such as composted manure and biochar, result in N_2_O emissions that are comparatively lower than raw manure and inorganic N fertilizers. Thus, a good understanding of the specific chemical composition of OFs is needed to minimize negative environmental impacts. Furthermore, published research shows that agroecological soil management can help in tightening the N cycle to reduce N losses through N_2_O emissions. Interestingly, long-term application of OFs and the buildup of soil C stocks may contribute to N retention as microbial or stabilized organic N while increasing the abundance of denitrifying microorganisms and thus reduce the production of N_2_O by favoring the completion of denitrification to produce N_2_.

Despite the recent interest in engineering the soil microbiome to increase agricultural sustainability, we still lack a fundamental mechanistic understanding of how OFs affect the N cycle in soils. Current literature investigating N_2_O emissions from OFs mostly focuses on the broad processes of nitrification and denitrification without providing a clear resolution on the specific microbial taxa and N transformations responsible for N_2_O emissions. Given that the N cycle is mostly composed of narrow processes, which are supported only by few microbial taxa [116], changes in the diversity of soil microorganisms imposed by OFs may have important consequences on N cycling and N_2_O production, as the rate of N_2_O production or reduction varies by taxa. High-throughput sequencing has been instrumental in revealing how fertilizer management practices drive microbial diversity and composition, but due to the large uncertainty in the physiology and N_2_O production rates of key N cycling microorganisms, assessment of microbial functions may be more important [30,117]. Thus, future research needs to determine if the structure can be used to predict microbial function, and a multi-omics approach can be used to establish key biochemical pathways and microbial taxa responsible for N_2_O production [117,118].

Another major challenge is integrating microbial processes into terrestrial ecosystems and biogeochemical models for the prediction of N_2_O emissions with different scenarios of land use [119]. Nitrous oxide production in soils is spatially variable due to the patchy distribution of N in soils and the existence of a great diversity of microenvironments and conditions within a soil profile. Temporal variability in N_2_O production is associated with changes in N inputs, soil moisture and oxygen availability, all of which are affected by OFs. This complicates accurately simulating and scaling up of these processes, resulting in large uncertainties in the model estimates of N_2_O responses to OFs.

## Figures and Tables

**Table 1:** Summary of the enzymes and microbial domains involved in the different steps of the N cycle.

Transformation	Enzyme	Gen	Microorganism
NH_4_^+^ -> NH_2_OH	Ammonia monooxygenase	*amoA*	Bacteria and Archaea
NH_2_OH -> NO	Hydroxylamine oxidoreductase	*hao*	Bacteria
NH_2_OH -> N_2_O	Cytochrome P460	*cyt P460*	Bacteria
NO -> NO_2_^−^	Dinitrite reductase	*nirK*	Bacteria
NO_2_^−^ -> NO_3_^−^	Nitrite oxidoreductase	*nxr*	Bacteria
NO_2_^−^ -> NH_4_^+^	Periplasmic nitrite reductase	*nrf*	Bacteria
NO_3_^−^ -> NO_2_^−^	Membrane bound dissimilatory nitrate reductase	*narG*	Fungi, Bacteria
	Periplasmatic dissimilatory nitrate reductase	*nap*	Fungi, Bacteria
NO_2_^−^ -> NO	Cd_1_ nitrite reductase	*nirS*	Bacteria
	Copper nitrite reductase	*nirK*	Bacteria, fungi
NO -> N_2_O	Nitric oxide reductase	*norB*	Bacteria
	Quinol nitric oxide reductase	*qnorB*	Bacteria
	Cytochrome P450nor nitric oxide reductase	*P450nor*	Fungi
	Nitrosocyanin	*ncyA*	Bacteria
N_2_O -> N_2_	Nitrous oxide reductase	*nosZ Clade I, nosZ Clade II*	Bacteria, Fungi
	Nitrosocyanin	*ncyA*	Bacteria

**Table 2:** Effects of manure-based organic fertilizers on the main microbial enzymes involved in the processes of nitrification and denitrification, based on the literature reported in the main text. Upward pointing arrows designate a predominantly positive effect; downward pointing arrows designate a negative effect; question marks indicate that not enough is known to draw conclusions.

	*Nitrification*			*Denitrification*							
	*amo*	*hao*	*nxr*	*narG*	*nap*	*nirS*	*nirK*	*norB*	*nosZ*	*ncyA*	References
Manure	**↑**	?	?	**↑**	?	**↑**	**↑**	?	**↑**	?	[37,56,68,71,72,73]
Digestate	**↑**	?	?	**↑**	?	?	?	?	**↑**	?	[74]
Compost	**↑**	?	?	**↑**	?	**↑**	**↑**	↓ **↑**	**↑**	?	[70,75,76,77]
Vermicompost	**↑**	?	?	?	?	↓	↓	?	**↑**	?	[78]
Biochar	?	?	?	?	?	**↑**	**↑**	?	**↑**	?	[79]
